# The challenges of pregnancy management in pyridoxine nonresponsive homocystinuria: The Irish experience

**DOI:** 10.1002/jmd2.12233

**Published:** 2021-06-09

**Authors:** Caroline Hart, Jenny McNulty, Melanie Cotter, Fatima Al Jasmi, Ellen Crushell, Ahmad Ardeshir Monavari

**Affiliations:** ^1^ Paediatric Metabolic Department, Royal Belfast Hospital for Sick Children Belfast UK; ^2^ National Centre for Inherited Metabolic Disorders, Children's Health Ireland at Temple Street Dublin Ireland; ^3^ Paediatric Haematology, Children's Health Ireland at Temple Street Dublin Ireland; ^4^ Department of Pediatrics, Tawam Hospital Al Ain United Arab Emirates; ^5^ University College Dublin Dublin Ireland

**Keywords:** pyridoxine nonresponsive homocystinuria, pregnancy in inborn errors of metabolism

## Abstract

Many patients with inborn errors of metabolism, due to early diagnosis and improved management, are living longer with less disease burden. Several are now having families of their own. This poses challenges both for the metabolic control of the mother and potential secondary effects on the fetus, as well as the risk of inheriting the inborn error. Classical homocystinuria (HCU, OMIM 236200) is a rare multisystem condition with intellectual, skeletal, ocular, and thromboembolic complications. Ireland has included HCU in the National Newborn Bloodspot Screening Program since 1971. The European network and registry for homocystinurias and methylation defects (E‐HOD) guidelines outline the requirements for management and monitoring of this condition and associated complications. Pregnancy alone has many potential complications. When combined with an underlying condition such as HCU, which is prothrombotic and requires a highly medicalized diet, there are significantly increased risks to both mother and baby. Colleagues previously published an Irish case of maternal HCU with successful pregnancy outcome. We add five pregnancies to two women with classical HCU to the literature. We use these to highlight the importance of careful metabolic control and managing the predictable HCU associated risks during pregnancy and the postpartum period. Our cases demonstrate the potential for healthy pregnancies in HCU and that this is best achieved with a motivated clinical team and good patient engagement. Only small numbers of pregnancies in HCU have been reported and we are still learning best practice, but proactive management is essential, as in any inborn error of metabolism.

## 1 INTRODUCTION

Many patients with inborn errors of metabolism, due to early diagnosis and improved management, are living longer with less disease burden. Several are now having families of their own. This poses challenges both for the metabolic control of the mother and potential secondary effects on the fetus, as well as the risk of inheriting the inborn error.

Classical homocystinuria (HCU, OMIM 236200), caused by a deficiency of cystathionine β‐synthase (CBS) in the metabolism of methionine, is a rare multisystem condition. Complications include intellectual impairment, skeletal disease (osteoporosis), ocular disorders (severe myopia and lens dislocation) as well as vascular (an increased risk of venous and arterial thromboembolic events) disease. The international prevalence of HCU varies with a clinically detected cases occurring in 0.82 per 100 000 and cases detected via newborn screening occurring on 1.09 per 100 000.^1^ In Ireland, the incidence is significantly higher, occurring in approximately 1 in 64  900 births, roughly equating to one baby being diagnosed with HCU via newborn screening annually.^2^ Ireland has included HCU in the National Newborn Bloodspot Screening Program (NNBSP) since 1971 and identified patients are treated at the National Centre for Inherited Metabolic Disorders (NCIMD) at Children's Health Ireland (CHI) at Temple Street. Careful adherence to a regimen of a strict diet with reduced natural methionine (a prescribed number of methionine exchanges where 1 exchange is equivalent to 1 g of natural protein and 25 mg methionine is used at NCIMD), methionine free amino acid mixture/supplements, low protein foods and medication is required to achieve good control and avoid or minimize complications. The European network and registry for homocystinurias and methylation defects (E‐HOD) guidelines^3^ outline the requirements for management and monitoring, including for complications. At the NCIMD, we have traditionally used free homocysteine (fHcy), the nonprotein bound fraction,^4^ for monitoring biochemical control and have more recently added total homocysteine (they). With consistent maintenance of fHcy <11 μmoL/L, equating to a tHcy <120 μmoL/L,^3^ our patients have not encountered complications.^2^ Pregnancy has many potential complications and combined with an underlying condition such as HCU, which is prothrombotic and requires a highly medicalized diet, there are significantly increased risks to both mother and baby. Previously Yap and Levy have documented pregnancy outcomes in HCU and to this we add five pregnancies to two women with classical pyridoxine nonresponsive HCU.^5^, ^6^, ^7^

### 1.1 Patient A

Our first patient is a nonconsanguineous Irish woman who was diagnosed via the NNBSP with pyridoxine nonresponsive HCU. Later genetic testing demonstrated homozygosity for the classical HCU mutation in the CBS gene, c.919G > A (p.Gly307Ser). A standard HCU diet, vitamins B6, B12, folic acid and monitoring was initiated in the neonatal period and in infancy and childhood good compliance was achieved with satisfactory biochemical control. However, adolescence proved more challenging with variable attendance at clinic and engagement with the diet, therapeutic monitoring, and medication. This required a period of increased frequency of outpatient review and intensive education and therapeutic management, including the addition of betaine to increase the methionine tolerance. Following these measures satisfactory biochemical control was achieved intermittently. She completed third level education and was in full‐time employment.

**GRAPH 1:**
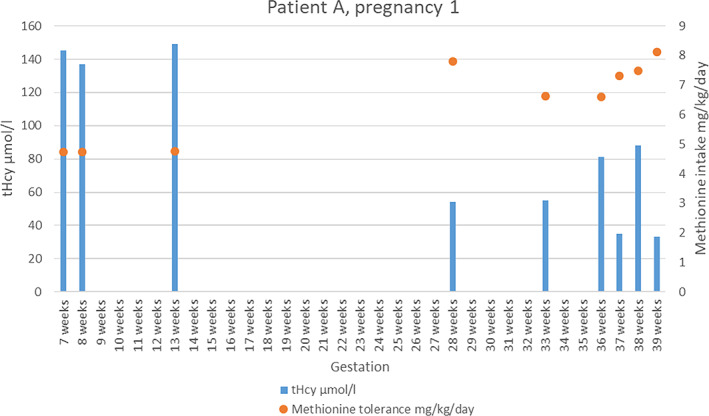
Total homocysteine level and methionine tolerance for patient A, pregnancy 1

Patient A developed osteoporosis of the lumbar spine and neck of femur demonstrated on dual energy X‐ray absorptiometry (DEXA) scans with a wrist fracture, which occurred following a simple fall at 18 years old. She was intermittently vitamin B12 deficient requiring parenteral supplementation. At 24 years of age, she experienced bilateral lens dislocations and cataract formation. Lens replacements were scheduled, but these were deferred as she became pregnant with her first child at 28 years old. A clear baseline methionine tolerance was difficult to establish due to longstanding challenges with dietary adherence (see Supplementary Table 1 for full dietary and biochemistry details during pregnancy). Her prescribed daily folate (5 mg) was regularly taken but her pyridoxine (100 mg) and oral B12 were not, as the patient found these difficult to tolerate. Initially, she was commenced on aspirin, but this was changed to low‐molecular weight heparin (LYMPH), which was continued until 6 weeks postpartum. Vitamin B12 injections were administered, along with re‐instatement of pyridoxine and increase in folate to 10 mg per day. During the pregnancy, there was improvement in engagement with more frequent clinic visits and a significant increase in contact with the dietetic team. However, dietary control remained challenging despite initial progress, with variable methionine exchanges being taken and low amino acid mixture/supplement intake even after a variety of options were trialed. Consistent medication tolerance also proved difficult. Overall, tHcy levels were satisfactory (see Graph 1) and the maximum number of methionine exchanges tolerated was 25 (equating to 25 g of daily natural protein and 8.11 mg/kg/day of methionine), from a baseline of approximately 12 (prepregnancy weight not available for mg/kg/day calculation). All antenatal scans were normal and there was close communication with the obstetric team. A healthy baby boy was delivered without complication and high‐risk newborn screening for HCU was negative. The patient remained well post‐delivery and continued LMWH for 6 weeks postpartum, although issues with therapeutic compliance later re‐emerged. Eye surgery was carried out the following year.

**GRAPH 2:**
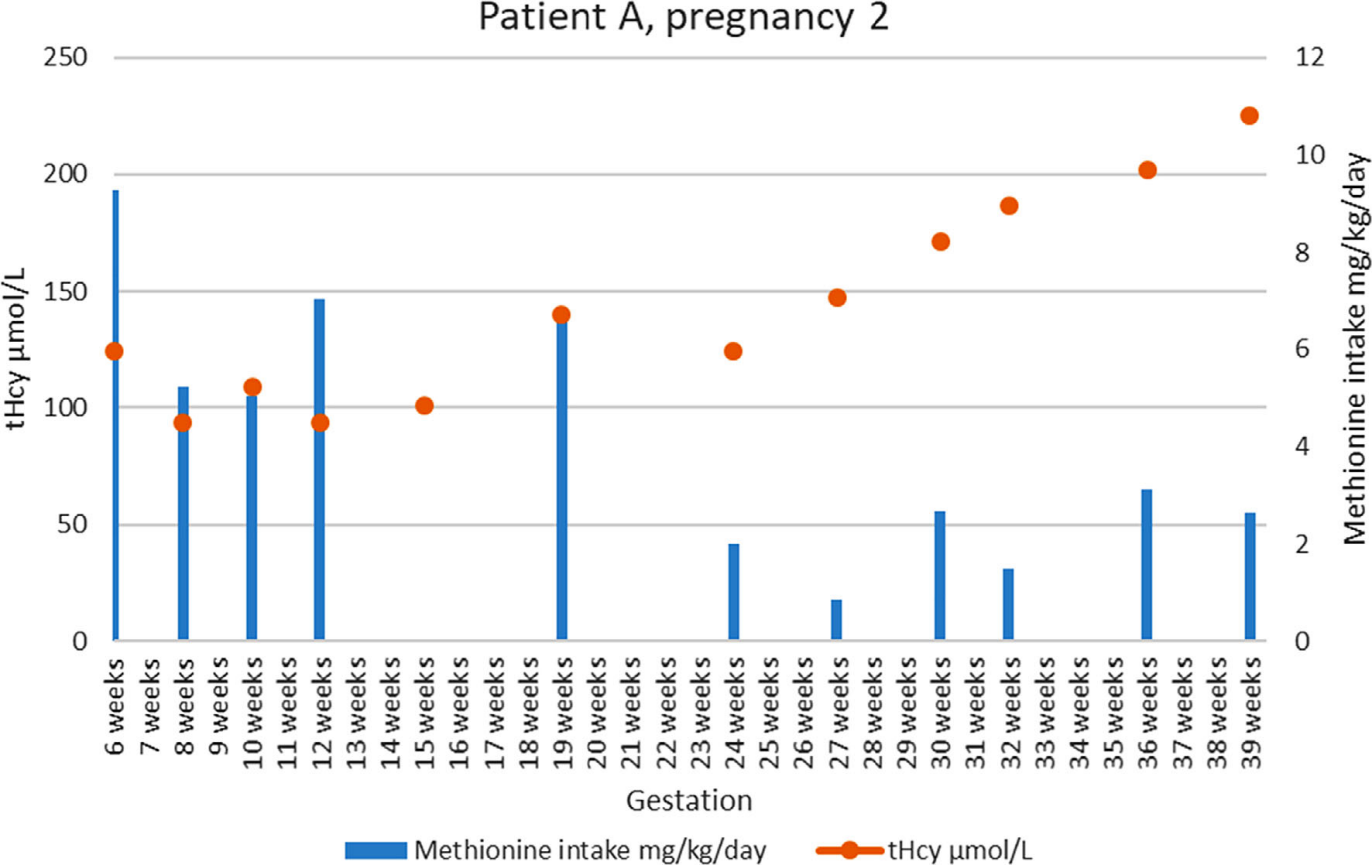
Total homocysteine levels and methionine intake for patient A in pregnancy 2

The patient became pregnant again the next year and was reviewed within the first few weeks when it was noted that tHcy levels were high (193 μmoL/L) and she was taking excessive dietary methionine but compliance with her amino acid mixture/supplement had improved. LMWH was commenced and HCU medications including pyridoxine and B12 were reintroduced, although tolerance varied throughout the pregnancy. Most tHcy and fHcy levels were within the target range and regular levels were supplied (see Graph 2). The maximum methionine exchanges reached in this pregnancy was 29 (equivalent to 29 g/day natural protein and approximately 10.82 mg/kg/day methionine) and a healthy baby boy was delivered at term, again screening negative for HCU. Unfortunately, the baby became unwell with a febrile illness unrelated to maternal HCU at 2 weeks of age and required hospital admission. During this time, the patient discontinued her postpartum LMWH just after 2 weeks, and subsequently she developed a pulmonary embolus (PE) at 11 weeks postpartum. No tHcy or fHcy levels are available from this time.

The patient was treated acutely and made a full recovery following the PE but was advised that future pregnancies would carry a significant thrombotic risk. Following a subsequent posterior sagittal sinus thrombosis at 37 years and she is now on warfarin. Both children remain healthy.

### 1.2 Patient B

Our second patient, a nonconsanguineous Irish woman, was also diagnosed with classical HCU through NNBSP, which was later genetically confirmed with homozygous c.919G > A (p.Gly307Ser) mutations in the CBS gene. She was on a protein restricted diet with amino acid mixture/supplements from diagnosis. Throughout childhood and adolescence, she exhibited good dietary and medication compliance with satisfactory biochemical control. There had never been any developmental concerns and she achieved third level education, going on to work in information technology with frequent long‐distance business travel. During her early years, she experienced significant myopia, although there was a family history of the same, had normal bone mineral density into adulthood and did not experience any thromboembolic events.

Prepregnancy planning with the hematology team determined LMWH from the date of a confirmed pregnancy would be appropriate. Prepregnancy natural protein tolerance was 9‐12 methionine exchanges (9‐12 g of natural protein), approximating to 225‐300 mg methionine per day (no weight available for mg/kg/day calculations). She attended outpatient review at the start of the second month of her first pregnancy at 32 years of age, having already been commenced on LMWH by her GP. Her pyridoxine dose was revised to 100 mg per day (previously 400 mg), she was already taking folic acid 800 μg per day and was on a pregnancy multivitamin which was then discontinued (as sufficient vitamins were supplied by amino acid mixture/supplement). Her LMWH was increased to 40 mg once daily by hematology service at 6 weeks gestation. Dietary compliance was good with satisfactory tHcy and fHcy levels and synthetic amino acid mixture/supplement provision of just under 1 g/kg/day protein equivalent. Unfortunately, the patient suffered a first trimester miscarriage but did not require surgical intervention.

**GRAPH 3:**
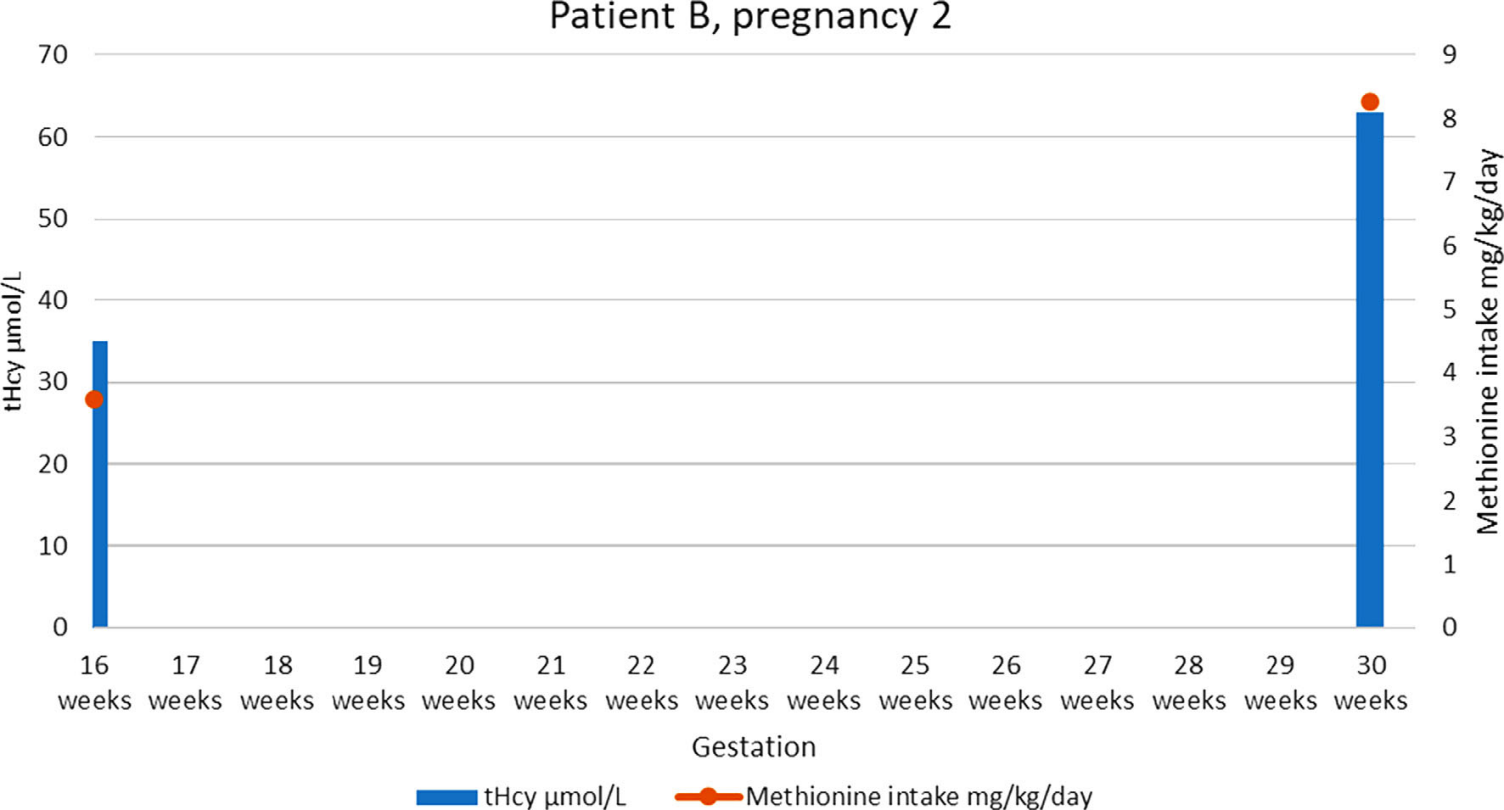
Total homocysteine levels and methionine intake for patient B, pregnancy 2. NB no prepregnancy weight available for baseline calculations but ranged from 9 to 12 methionine exchanges per day prepregnancy to a maximum of 23 per day, then back to 12 postpartum when breastfeeding

A few months later the patient returned to clinic at 9 weeks gestation in her second pregnancy. She was well at this time with good metabolic control (see Graph 3). Again, LMWH had been commenced by her GP and she was linked in with obstetric services abroad due to her employment. At this time, she remained on folate 800 μg per day and pyridoxine 100 mg per day alongside her amino acid mixture/supplement. A second outpatient review took place at 16 weeks gestation and no acute issues were raised. A plan was provided for the local obstetric team to commence IV fluids during delivery and avoid nitric oxide for any required anesthesia. Metabolic control was ensured through on‐going biochemical monitoring of fHcy in Dublin and tHcy in a metabolic center close to where she was living and working abroad, alongside dietetic input and clinical metabolic assessments. The maximum natural methionine exchanges achieved during the pregnancy was 23 (23 g of natural protein), approximating to 8.27 mg/kg/day methionine (see supplementary Table 2). A healthy baby girl was delivered normally at term and was successfully breastfed.

**GRAPH 4:**
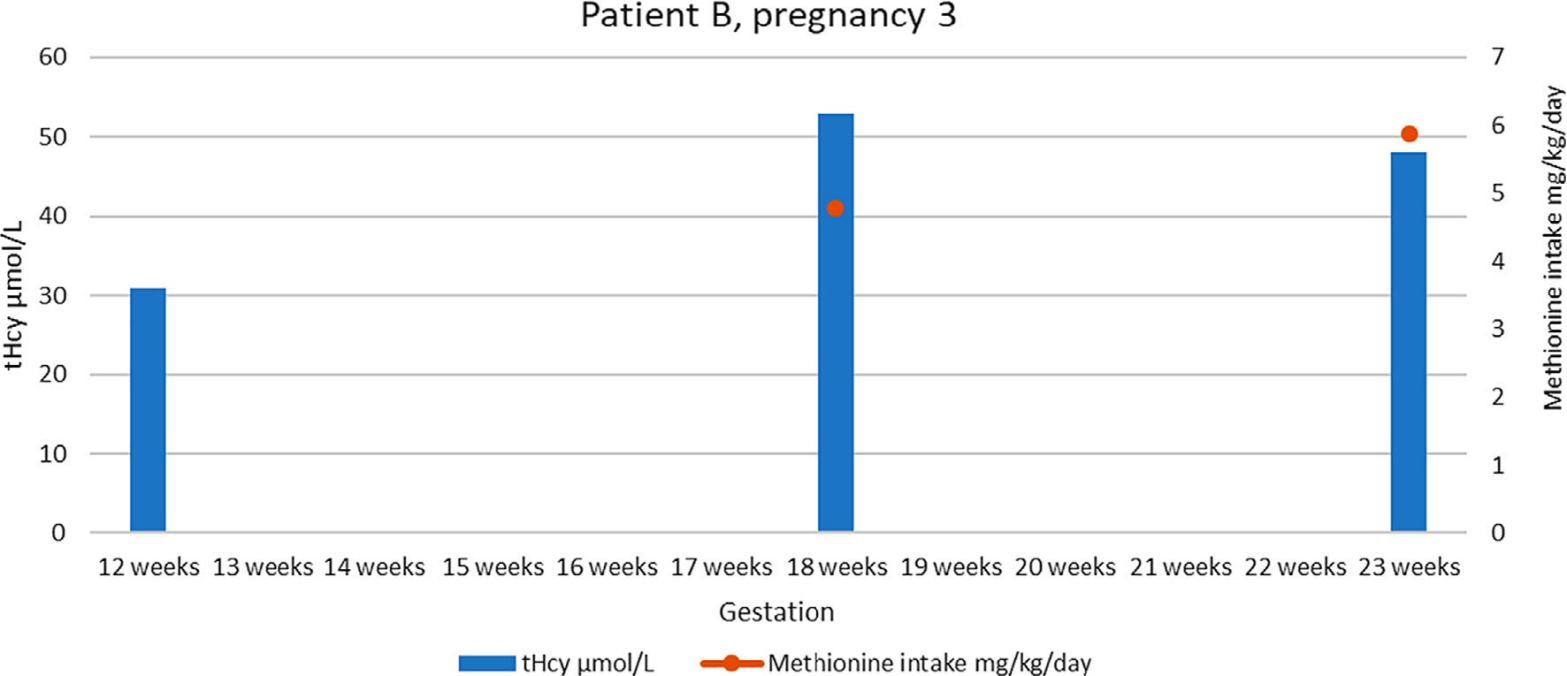
Total homocysteine levels and methionine intake for patient B, pregnancy 3

At the next outpatient review the following year the patient was 18 weeks pregnant, having had a normal antenatal scan and been commenced on LMWH by obstetrics at her local center abroad. Her medications and amino acid mixture/supplements were unchanged from her previous pregnancy. Again, good compliance and metabolic control was achieved throughout this pregnancy (see Graph 4) and a healthy term baby boy was delivered without complication. The patient continued on LMWH for a number of weeks postpartum for each pregnancy and neither infant was diagnosed with HCU. She successfully breastfed both babies and therefore required higher natural protein, and therefore methionine, intake during this period. Following cessation of breastfeeding, the patient returned to her baseline methionine tolerance. See supplementary Table 2 for a full summary of methionine intake and available biochemistry during pregnancy.

## 2 DISCUSSION

Previous reports of pregnancy in HCU have described various management approaches. In 2001, Yap and colleagues described the case of an Irish patient with pyridoxine unresponsive HCU with successful maternal and infant outcomes.^5^ That patient more than doubled their methionine tolerance during the pregnancy and achieved fair biochemical control, which had been previously suboptimal. Their case emphasized the importance of close monitoring and active measure to prevent thromboembolic complications.^5^

The largest cohort of pregnancies in HCU was reported by Mudd and colleagues, although the majority of these were in pyridoxine responsive women.^7^ They described 11 pregnancies in classical HCU, 8 of which resulted in live born infants and they noted the various, mainly postpartum maternal complications in all categories of HCU.^7^ In 2002, Levy and colleagues described their experience with 12 successful pregnancies (plus two early miscarriages) in 11 women, five of whom were pyridoxine nonresponsive.^6^ Their study showed infrequent pregnancy complications, but the dietary and medical management was quite varied, and the majority did not receive anticoagulation. They suggested that the high plasma Hcy levels seen in the postpartum period may be reflected by a particularly increased thrombotic risk.^6^ Vilaseca et al reported their experience with a woman with pyridoxine nonresponsive HCU who had three pregnancies with two healthy children and one miscarriage.^8^ Much like one of our cases, this woman had multiple baseline complications including eye and bone disease but unlike our cohort this patient was diagnosed late at 1 year of age with ectopia lentis. Although the importance of anticoagulation was emphasized in this case report, a combination of acetylsalicylic acid (aspirin) and heparin was used instead of LMWH.^8^ A more recent case report in 2006 also highlights the importance of anticoagulation, using first aspirin and then LMWH, and akin to patient A, their patient achieved good biochemical control despite prenatal poor control.^9^

The E‐HOD guidelines specifically recommend the use of LMWH during the third trimester, and perhaps throughout pregnancy, up until at least 6 weeks postpartum.^3^ They also go on to state that in the presence of additional thrombosis risk factors women should have LMWH prescribed for the whole pregnancy. E‐HOD noted that LMWH was the most widely used method of anticoagulation but that others were in use.^3^ Indeed, there is much experience with anticoagulation in obstetric practice. The Royal College of Obstetricians and Gynaecologists (RCOG) guideline on this issue stratifies women into at risk categories for venous thromboembolism (VTE) and uses this to determine how long to employ anticoagulation.^10^ Their anticoagulant of choice is LMWH and their standard duration of treatment in the postnatal period is 6 weeks, even in the case of prior VTE at which point those patients would re‐establish their maintenance treatment.^10^ It is logical therefore that the same practice be applied to HCU patients postpartum, although perhaps if there were issues with biochemical control and elevated fHcy or tHcy levels persist there could be merit in adapting this on a case by case basis and advice from hematology colleagues would be beneficial. This again is in line with the RCOG guideline, stating to use LMWH in high risk groups “for up to 6 weeks or until the additional risk factor/s is/are no longer present.”^10^ In patient A's case where VTE occurred at 11 weeks postpartum, there was early discontinuation of LMWH and it is unclear how strict their biochemical control was during this period, although we know baseline control was sub‐optimal. Therefore, it would be difficult to strongly recommend adaptations to the current guidance on the postpartum duration of LMWH in this high‐risk group based on this single case. However, our case highlights the need for close biochemical monitoring in the postpartum period until at least the end of the 6‐week window of increase VTE risk and if biochemical stability it not reached the possibility of extending LMWH treatment should be discussed with hematology. Also of note, both our patients received LMWH from early pregnancy based on hematology advice and this has also been a practice reported previously^9^ though current guidelines suggest only consideration of this.^3^ Based on our experience, we would recommend LMWH therapy from pregnancy confirmation and early hematology input. In addition to the use of LMWH, from our experience, we recommend ensuring adequate hydration during labor by pre‐emptively commencing IV fluids with the aim of reducing the risk of thrombosis, much like the E‐HOD guidance for the management of undercurrent illness and surgery.^3^ Modifying this risk are of importance to both mother and infant.

It is worth noting that there is no evidence to suggest an increased risk of miscarriage in HCU.^3^ This is despite it inferring a risk of VTE and that prothrombotic conditions tend to heighten the risk of pregnancy loss. In our case series, and those quoted above, early miscarriage was a feature, although this is likely to be reflective of the background population risk.

Both of our patients demonstrated an increased methionine, and therefore natural protein, intake and reduced tHcy during the third trimester, although the trend is less clear for patient B due to split site monitoring. This is a phenomenon reported previously^5^, ^9^ and is similar to the increased phenylalanine tolerance experienced by mother's with PKU in the later stages of pregnancy also.

As HCU control is reliant on adherence to a strict medicalized diet, deficiencies in nutrition of the mother and therefore the unborn infant could occur if dietary control is not adhered to. This relates not just too natural protein intake, the number of methionine exchanges taken but also ensuring appropriate vitamin, and mineral supplementation is achieved. Amino acid mixtures/supplements are fortified to meet dietary requirements and consistent tolerance of these is needed as well as considering the specific micronutrient needs of the expectant mother.

The cases presented here highlight the importance of careful metabolic control and managing the risks associated with HCU during pregnancy and the postpartum period. Both of our patients demonstrate the potential for healthy pregnancies in this condition and that this is best achieved with a motivated clinical team and an engaged patient. With such small numbers of pregnancies in HCU, we are still learning best practice and pre‐emptive management is essential, as in any inborn error of metabolism, with particular consideration needed in three phases: preconception, pregnancy, and the postpartum period.^11^

### 2.1 Learning points

Previous reports have shown that in the Irish newborn screened adult patients with pyridoxine nonresponsive HCU, most have no or only minor complications of HCU and are leading healthy lives.

Challenges with metabolic control can still arise at any stage in this condition and require a multidisciplinary approach with patient engagement.

When considering pregnancy in HCU early consultation with hematology services and close communication with the obstetric team is important, so that clear plans can be established to minimize thromboembolic risk.

Pregnancy poses increased risk to women with HCU and pre‐emptive management is crucial to prevent harm to both mother and baby, with a particular focus required on nutrition and the risk of thromboembolism.

## CONFLICT OF INTEREST

The authors declare no conflicts of interest.

## INFORMED CONSENT

All procedures followed were in accordance with the ethical standards of the responsible committee on human experimentation (institutional and national) and with the Helsinki Declaration of 1975, as revised in 2000 (5). Informed consent was obtained from all patients for being included in the study. Written informed consent was obtained from the patients included in this article and their identifying information is not included in this article.

## ANIMAL RIGHTS

This article does not contain any studies with human or animal subjects performed by any if the authors.

## AUTHOR CONTRIBUTIONS

Caroline Hart drafted the article, reviewed the literature and revised the manuscript, also acting as guarantor. Jenny McNulty critically reviewed the article and provided a dietetic perspective. Melanie Cotter reviewed the article and provided hematological advice. Fatima Al Jasmi revised the article content, as did Ellen Crushell. Ahmad Ardeshir Monavari created the article concept and critically appraised each draft.

## Supporting information

**Supplementary Table 1** Dietary management and biochemistry results for Patient A
**Supplementary Table 2:** Dietary management and biochemistry results for Patient B
