# Supplementary material for: The challenges of pregnancy management in pyridoxine nonresponsive homocystinuria: The Irish experience
Source: JIMD Rep. 2021 Jun 9;61(1):34–41. doi: 10.1002/jmd2.12233 (PMC8411100; doi:10.1002/jmd2.12233)
Supplement: Supplementary file 1 — Supplementary Table 1 Dietary management and biochemistry results for Patient A Supplementary Table 2: Dietary management and biochemistry results for Patient B [file JMD2-61-34-s001.docx]

**Supplementary table 1:** Dietary management and biochemistry results for Patient A

|  | tHcy (µmol/l) | fHcy (µmol/l) | Methionine (µmol/l) | Free Cystine (µmol/l) | Weight (kg) | Natural protein intake (g/day)  NB. 1g natural protein = 1 methionine  exchange = 25mg methionine | Methionine intake (mg/kg/day) | Amino acid mixture/supplement |
| --- | --- | --- | --- | --- | --- | --- | --- | --- |
| Baseline |  |  |  |  |  |  |  | 4½ sachets per day met maxamum (approx. 1/kg/day protein equivalent)- variable tolerance throughout and different options trialled e.g.  HCU cooler (transitioned to this during second pregnancy) |
| Pre-pregnancy | 181 | 16 | 52 | 27 | - | 12 | - |  |
| Pregnancy 1 |  |  |  |  |  |  |  |  |
| Gestation |  |  |  |  |  |  |  |  |
| 5 weeks | - | - | 15 | 37 | - | 10 | - |  |
| 6 weeks |  | 16 | 66 | 19 | 63.4 | 8 | 3.15 |  |
| 7 weeks | 145 | 18 | 62 | 19 | - | 12 | 4.73 |  |
| 8 weeks | 137 | 6 | 75 | 11 | - | 12 | 4.73 |  |
| 9 weeks | - | - | - | - | 63.2 | - | - |  |
| 13 weeks | 149 | 14 | 58 | 20 | - | 12 | 4.75 |  |
| 16 weeks | - | 8 | 60 | 27 | 64.2 | 12 | 4.67 |  |
| 20 weeks | - | <5 | 41 | 25 | - | 14 | 5.45 |  |
| 25 weeks | - | - | 29 | 32 | - | 18 | 7.01 |  |
| 28 weeks | 54 | <5 | 26 | 36 | - | 20 | 7.79 |  |
| 33 weeks | 55 | - | 30 | 39 | - | 17 | 6.62 |  |
| 35 weeks | - | - | - | - | 75.8 | - | - |  |
| 36 weeks | 81 | - | 37 | 38 | - | 20 | 6.60 |  |
| 37 weeks | 35 | - | 21 | 54 | 77 | 22-23 | 7.31 |  |
| 38 weeks | 88 | 5 | 44 | 37 | - | 23 | 7.47 |  |
| 39 weeks | 33 | - | 22 | 57 | - | 25 | 8.11 |  |
| Pregnancy 2 |  |  |  |  |  |  |  |  |
| Gestation |  |  |  |  |  |  |  |  |
| 6 weeks | 193 | 22 | 38 | 12 | 67 | 14-18 | 5.97 |  |
| 8 weeks | 109 | 7 | 30 | 22 | - | 12 | 4.48 |  |
| 10 weeks | 105 | 5 | 27 | 30 | - | 14 | 5.22 |  |
| 12 weeks | 147 | 11 | 49 | 27 | - | 12 | 4.48 |  |
| 15 weeks | - | 8 | 46 | 29 | - | 13 | 4.85 |  |
| 19 weeks | 143 | 15 | 69 | 21 | - | 18 | 6.72 |  |
| 23 weeks | - | 21 | 65 | 18 | - | 15 | 5.60 |  |
| 24 weeks | 42 | <5 | 19 | 32 | - | 16 | 5.97 |  |
| 27 weeks | 18 | - | 7 | 56 | - | 19 | 7.09 |  |
| 30 weeks | 56 | <5 | 38 | 34 | - | 22 | 8.21 |  |
| 32 weeks | 31 | - | 19 | 48 | - | 24 | 8.96 |  |
| 36 weeks | 65 | <5 | 38 | 40 | - | 26 | 9.70 |  |
| 39 weeks | 55 | - | 16 | 53 | - | 29 | 10.82 |  |

NB. Methionine intake as mg/kg /day calculated using most recent available weight in medical records. Where range of exchanges taken the mean was used for calculations.

**Supplementary table 2:** Dietary management and biochemistry results for Patient B

|  | tHcy(µmol/l) | fHcy (µmol/l) | | Methionine (µmol/l) | Free cystine (µmol/l) | | Weight (kg) | | | Natural protein intake (g/day)  NB. 1g natural protein = 1 methionine  exchange = 25mg methionine | Methionine intake (mg/kg/day) | | Amino acid mixture/supplement | |
| --- | --- | --- | --- | --- | --- | --- | --- | --- | --- | --- | --- | --- | --- | --- |
| Baseline |  |  |  | | |  | |  |  | | |  | | HCU LV x 3  (60g protein equivalent/  day) |
| Pre-  pregnancy | 89 | 6 | 53 | | | 36 | | - | 9-12 | | | - | |  |
| Pregnancy 1 |  |  |  | | |  | |  |  | | |  | |  |
| Gestation |  |  |  | | |  | |  |  | | |  | |  |
| 5 weeks | - | 6 | 39 | | | 33 | | - | 9-12 | | | - | |  |
| 7 weeks | 39 | 4 | 23 | | | 39 | | - | 9 | | | - | |  |
| Pregnancy 2 |  |  |  | | |  | |  |  | | |  | |  |
| Gestation |  |  |  | | |  | |  |  | | |  | |  |
| 16 weeks | 35 | - | 22 | | | 34 | | 69.55 | 10 | | | 3.59 | |  |
| 20 weeks | - | 5 | 33 | | | 26 | | - | Unknown | | | - | |  |
| 30 weeks | 63 | 4 | 32 | | | 63 | | - | Max 23 | | | 8.27 | |  |
| Post-partum (breast-feeding) | 70 | <3 | 31 | | | 39 | | - | 12 | | | - | |  |
| Pregnancy 3 |  |  |  | | |  | |  |  | | |  | |  |
| Gestation |  |  |  | | |  | |  |  | | |  | |  |
| 12 weeks | 31 | <3 | 14 | | | 22 | | - | 13 | | | - | |  |
| 14 weeks | - | 3 | 23 | | | 29 | | - | 13 | | | - | |  |
| 18 weeks | 53 | 3 | 28 | | | 27 | | 67.9 | 13 | | | 4.79 | |  |
| 23 weeks | 48 | <3 | 22 | | | 32 | | - | 16 | | | 5.89 | |  |

NB. Limited weight, biochemistry and dietary data available due to shared site management. Methionine/kg/day calculated on only available weights for pregnancies. Where range of exchanges taken the mean was used for calculations.
